# Combination therapy with aromatase inhibitors: the next era of breast cancer treatment?

**DOI:** 10.1038/sj.bjc.6603316

**Published:** 2006-08-22

**Authors:** A Leary, M Dowsett

**Affiliations:** 1 Academic Department of Biochemistry, Royal Marsden Hospital/Institute of Cancer Research, London, UK

**Keywords:** endocrine therapy, resistance, aromatase inhibitors, targeted therapies, signal transduction inhibitors

## Abstract

Long-term endocrine therapy with either aromatase inhibitors (AIs) or tamoxifen may lead to endocrine resistance and disease progression. Recent years have seen advances in our understanding of the complex biological mechanisms associated with resistance. Growth factor signaling pathways appear to be upregulated in hormone-resistant tumours and interact with oestrogen-receptor (ER) signaling, which remains functional even after long-term endocrine deprivation. Signaling through the human epidermal and insulin-like growth-factor receptor (HER and IGFR, respectively) pathways may promote ligand-independent ER gene transcription and stimulate growth factor signaling. Therapeutic agents that inhibit these signal transduction pathways, when combined with AIs, may offer breast cancer patients new hope for more robust, longer-term remissions. Preliminary data from phase II studies of combination therapies are encouraging. There is a large programme of ongoing randomised, controlled trials, the results of which should pave the way for integrating combination therapies into clinical practice. To identify which patients will respond best to particular combinations of treatments, biomarkers and gene expression profiles are being investigated as predictors of sensitivity or resistance. In time, breast cancer treatment will become truly individualised because physicians will be able to match patients with a variety of disease phenotypes to optimal combination therapies.

Aberrant oestrogen-receptor (ER) expression/functioning has been implicated in the development of over 70% of breast cancers, the most common malignancy among women in the US and in Europe. Many, but not all patients with ER-positive (ER+) disease respond to hormonal therapy (e.g., tamoxifen, aromatase inhibitors (AIs)), but a large number will ultimately develop resistance with long-term treatment. There is evidence that peptide growth factors and their downstream effectors may interact with ER signaling to influence the sensitivity of breast cancer cells to endocrine therapy, and thus may be important targets for novel treatments.

Combining the most effective oestrogen-deprivation therapies with new therapies that target oestrogen-independent signaling pathways may increase the efficacy of breast cancer treatment. Advances in methods of assessing biomarkers and gene expression profiles that predict response to treatment may in the future allow physicians to tailor novel combination therapies to individual patients. This review summarises current knowledge about the mechanisms of endocrine resistance, the scientific rationale for combination therapy with AIs, and the clinical promise of this new approach.

## OVERCOMING ENDOCRINE THERAPY RESISTANCE AND THE RATIONALE FOR COMBINATION THERAPY

Endocrine therapy is the treatment of choice for hormone-sensitive breast cancer; however, over time many patients become resistant as tumours develop the ability to escape the antiproliferative effects of endocrine therapy (Martin *et al*, 2003). Understanding the various mechanisms responsible for the development of resistance to oestrogen deprivation will identify new therapeutic strategies to enhance the efficacy of breast cancer treatment. Some of the mechanisms of endocrine resistance involve oestrogen hypersensitivity, upregulation of signal transduction pathways, and crosstalk between upregulated signal transduction pathways and the ER pathway.

### Oestrogen hypersensitivity

Even following the development of endocrine resistance, ER signaling may continue to play an important role in the proliferation of breast cancer. Biopsies of tumours from the majority of breast cancer patients who have relapsed on tamoxifen show a functional ER (Johnston *et al*, 1997), and women who have become refractory to tamoxifen respond to further endocrine manipulation with the ER*α* downregulator fulvestrant (Dowsett *et al*, 2005a), indicating that ER-mediated signaling remains functional even in the setting of endocrine resistance. *In vitro* studies with long-term oestrogen-deprived cells (LTED), a line of MCF7 breast cancer cells developed under oestrogen-deprived conditions, have shown that these breast cancer cells adapted to endocrine deprivation by becoming hypersensitive to oestradiol doses as low as 10^−12^ M (Martin *et al*, 2003, Santen *et al*, 2005). Data from this preclinical model suggest that resistance to AIs may be due to acquired hypersensitivity to low oestradiol levels.

### Crosstalk between ER and growth-signaling pathways

**Figure 1:**
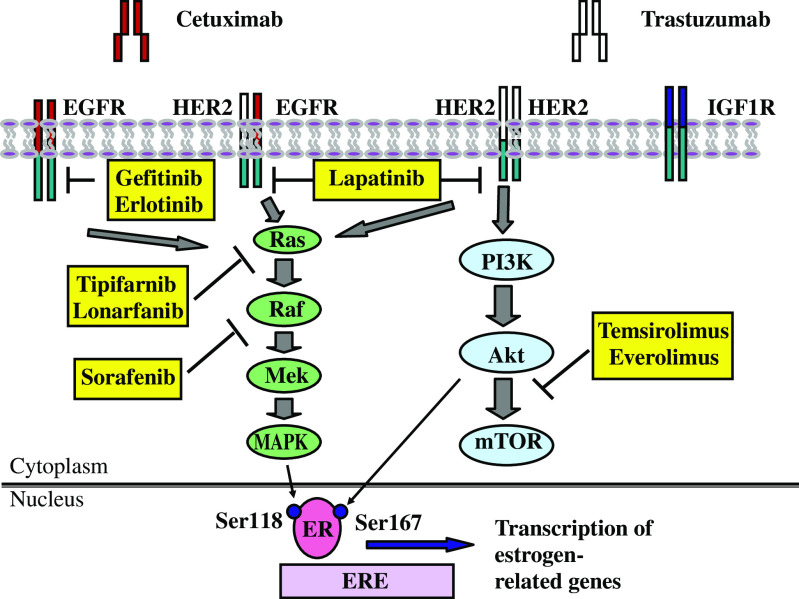
A model for endocrine resistance and its treatment: crosstalk between growth-factor receptor and ER pathways. Increased growth factor signaling may contribute to endocrine resistance by directly activating ER and leading to the transcription of oestrogen-related genes. Novel therapies can target a number of steps along this dysregulated signaling pathway and may therefore have an important role in the treatment of endocrine resistant breast cancer.

Adaptive upregulation of growth-signaling pathways leading to ligand-independent ER gene transcription may also lead to endocrine resistance (Figure 1). The epidermal growth-factor receptor (EGFR) and the human epidermal growth-factor receptor 2 (HER-2) become upregulated in response to endocrine deprivation or tamoxifen treatment (Knowlden *et al*, 2003; Martin *et al*, 2003) and are associated with disease progression, poor prognosis and resistance to tamoxifen (Kurokawa *et al*, 2000; Knowlden *et al*, 2003).

Activation of EGFR or HER2 stimulates two major intracellular kinase signaling cascades – the ras-raf-mitogenic-activated protein kinase (MAPK) pathway and the phosphatidylinositol 3-kinase (PI3K)/Akt pathway. These pathways activate downstream effectors, which phosphorylate and activate ER*α* and its coactivators (e.g., A1B1). In turn, the activation of ER*α* stimulates the production of growth factors (e.g., TGF*α*) and growth-factor signaling, creating a synergistic, self-reinforcing TGF*α*-ER autocrine loop (Knowlden *et al*, 2003). Over time, ER signaling may become dependent on these alternate signaling pathways, allowing cells to bypass normal endocrine responsiveness.

Recent evidence confirms the causal link between activated MAPK and PI3K pathways and the development of endocrine resistance. Studies using the previously mentioned LTED cells as well as TAMR cells, a tamoxifen-resistant cell line generated by continuous growth of MCF7 cells in tamoxifen containing media for 4–6 months, show adaptive upregulation of EGFR and HER2 with resulting activation of the downstream effector, MAPK (Knowlden *et al*, 2003; Martin *et al*, 2003). In turn, activated MAPK may phosphorylate the ER at Ser118 leading to ligand-independent transcription of oestrogen responsive genes (Chen *et al*, 2002). Blocking EGFR signaling (using gefitinib) reduces active MAPK and inhibits proliferation of tamoxifen resistant breast cancer cells (Knowlden *et al*, 2003), while the MAPK inhibitor, U0126, partially restores tamoxifen sensitivity (Kurokawa *et al*, 2000). Hypersensitive LTED cells also show increased levels of Akt and mTOR (mammalian target of rapamycin), and dual inhibition of MAPK and mTOR reverses oestrogen hypersensitivity (Santen *et al*, 2005). Similarly, increased IGF1R signaling has been observed in response to long-term oestrogen deprivation (Martin *et al*, 2005).

In summary, growth factor signaling, via EGFR, HER2 or IGF1R, becomes upregulated with the development of endocrine resistance and may lead to ligand-independent ER gene transcription. The synergistic interaction between the ER pathway and growth-signaling pathways promotes hormone resistance and disease progression. Various signal transduction inhibitors (STIs), that target the extracellular and intracellular domains of EGFR, HER2 and IGFR, are in development. These new therapies, in combination with potent ER inhibitors, may prevent or delay endocrine resistance and improve outcomes for women with ER+ breast cancer.

## COMBINATION THERAPY WITH AN AI: PRECLINICAL AND CLINICAL DATA

### Growth-factor receptor inhibitors

#### EGFR inhibitors

Gefitinib and erlotinib block the intracellular tyrosine kinase (TK) domain of EGFR by competitively binding to the receptor's adenosine triphosphate (ATP) site. *In vitro* data have shown that TAMR and LTED cells are more sensitive to gefitinib than the wild-type endocrine-sensitive MCF-7 cells (Knowlden *et al*, 2003; Martin *et al*, 2003).

Clinical studies of gefitinib and erlotinib monotherapy in unselected breast cancer patients have generally produced disappointing results; however, a phase II study of gefitinib monotherapy in ER+ tamoxifen-resistant patients demonstrated antitumour activity (Robertson *et al*, 2003), confirming the *in vitro* observations that gefitinib's efficacy may be primarily in the endocrine-resistant setting. Neoadjuvant single-agent gefitinib and gefitinib combined with the AIs anastrozole effectively reduced the size of breast tumours and levels of ER phosphorylation in previously untreated patients with ER/EGFR positive disease, with the combination treatment outperforming gefitinib alone in terms of reduction in tumour proliferation rate as measured by Ki67 (Polychronis *et al*, 2005). In contrast another phase II study of anastrazole in combination with gefitinib in patients with ER+ advanced disease who had previously failed hormonal therapy showed a lack of robust antitumour activity (Mita *et al*, 2005). However the inclusion criteria for these two studies differed. Mita's trial did not screen for EGFR. In contrast, Polychronis' study required both ER and EGFR positivity as inclusion criteria which may have enriched the patient population and could explain their positive results. There are a number of phase II trials underway evaluating the combination of gefinitib and AIs in metastatic breast cancer (Table 1).

#### HER2 inhibitors

15–20% of breast cancers overexpress HER2 and data from neoadjuvant trials have shown that these tumours may be relatively resistant to tamoxifen but remain sensitive to AIs (Ellis *et al*, 2001). Thus, for hormone-resistant breast cancer, particularly for ER+ tumours that overexpress HER-2, combining AIs with HER-2 inhibitors may be a more effective treatment approach. It is a common misconception that most HER-2+ tumours are ER-negative. While a greater proportion of ER− than ER+ tumours are HER2+, the greater absolute number of ER+ tumours results in similar proportions of HER2+ tumours being ER+ and ER−. This is illustrated by 50% of patients enrolled in the recent adjuvant trastuzumab clinical trials being either ER+ or progesterone-receptor+.

Trastuzumab binds to the extracellular domain of HER-2, reduces downstream MAPK/ERK1/2 signaling, and at least partially reverses tamoxifen resistance *in vitro* (Kurokawa *et al*, 2000). Preliminary findings from phase II clinical trials of letrozole and trastuzumab in patients with hormone-sensitive, HER-2–positive metastatic breast cancer revealed that the combination was well tolerated and had a clinical benefit rate of 50% with durable responses in 25% of the patients (Marcom *et al*, 2005 ASCO).

There are several ongoing nonrandomised phase II trials examining the efficacy of combining trastuzumab and AIs, and a phase III randomised, controlled trial comparing anastrozole with and without trastuzumab (Table 1).

#### Combined EGFR and HER2 inhibitors

Lapatinib is a dual oral TK inhibitor (TKI) of EGFR and HER-2 and thus may have a greater anticancer effect than therapies that target only one of these receptors. The combination of lapatinib and tamoxifen effectively blocked cell cycle progression *in vitro* and showed significant antitumour activity *in vivo* in tamoxifen-resistant tumour xenografts (Chu *et al*, 2005).

Treatment with single-agent lapatinib resulted in impressive partial response and stable disease rates of 28 and 40%, respectively, among 60 patients with previously untreated HER-2+ metastatic breast cancer (Gomez *et al*, 2005), and a large phase III trial exploring the efficacy of lapatinib in combination with letrozole is underway (Table 1). The combination of letrozole with AEE788, a dual TKI of EGFR/HER-2 and inhibitor of the vascular endothelial growth factor (VEGF) receptor is also being investigated.

Imatinib mesylate, a TKI of platelet-derived growth factor (PDGF) and c-Kit, has demonstrated an antiproliferative effect in a number of breast cancer cell lines but is ineffective as monotherapy in metastatic breast cancer (Modi *et al*, 2005). Small nonrandomised phase II trials are exploring the efficacy of combining imatinib with letrozole.

### mTOR inhibitors

The PI3K/Akt pathway interacts with ER*α* and is often aberrantly upregulated in breast cancers, therefore a promising therapeutic strategy is to inhibit the molecular target of rapamycin (mTOR), a key downstream effector of the PI3K enzyme. Preclinical studies have supported the activity of rapamycin analogues CCI 779 (temsirolimus) and RAD-001 (everolimus) in breast cancer cells with activated Akt and showed that the addition of everolimus reduced proliferation by a further 50% compared with letrozole alone (Farmer *et al*, 2005).

Several randomised, controlled trials are underway to determine the efficacy and safety of letrozole with temsirolimus or everolimus in ER+ breast cancer (Table 1). One of these studies utilises a two-stage design whereby biomarkers of response to the combination of letrozole and everolimus will be identified in a phase II neoadjuvant trial, and based on these biomarkers, patients will be randomised to letrozole alone or letrozole and everolimus as first-line therapy in a subsequent phase III trial. Unfortunately despite encouraging preclinical and early phase II data (Carpenter *et al*, 2005), HORIZON, a large phase III trial exploring the efficacy of the combination of letrozole and temsirolimus in metastatic ER+ breast cancer was recently terminated after an independent interim analysis reported that the combination showed no benefit over letrozole alone.

### Farnesyl transferase inhibitors

The ras proto-oncogene can activate downstream TK substrates (e.g., MAPK, MAPK/ERK kinase (MEK)) that influence cell proliferation, survival and apoptosis and has been implicated in breast cancer (O'Regan and Khuri, 2004). Farnesyl transferase inhibitors (e.g., tipifarnib, lonafarnib) were developed to inhibit tumour growth by blocking farnesylation, the first step in ras activation.

Both *in vitro* and *in vivo* studies have shown that tipifarnib inhibits the growth of MCF-7 breast cancer tumours (O'Regan and Khuri, 2004), and a subsequent phase II study reported clinical benefit in 24% of women with endocrine-resistant metastatic breast cancer (Johnston *et al*, 2003).

The modest clinical activity of FTI monotherapy and results from an animal study, in which the combination of tipifarnib and tamoxifen resulted in significantly greater tumour regression than either tamoxifen or tipifarnib alone (Johnston *et al*, 2002), raised the possibility that FTIs may be more effective in combination with endocrine treatment. A number of phase II randomised, controlled trials are currently exploring the potential efficacy of combining tipifarnib or lonafarnib with AI therapies (Table 1). However, a recently reported phase II randomised study of letrozole±tipifarnib in 121 women with tamoxifen resistant advanced breast cancer demonstrated no benefit to the combination compared to letrozole alone (Johnston *et al*, 2005). The reasons for these disappointing results are unclear, however, they may stem from the fact that the exact targets of FTIs are still poorly understood as many intracellular proteins aside from ras require farnesylation. Also, ras may be able to escape the inhibitory effect of FTIs by developing compensatory geranylgeranylation, which may suggest a role for dual geranylgeranyl transferase-1/farnesyl transferase inhibitors.

### Antiangiogenic agents

In order to grow beyond the size of 1–2 mm, tumours need to develop their own vasculature. This process, known as angiogenesis and principally mediated via tumoural secretion of VEGF, is critical to tumour survival and has therefore emerged as an attractive anticancer strategy. Bevacizumab, a humanised monoclonal antibody directed against all active isoforms of VEGF, does not appear to be effective as a single agent in breast cancer but may enhance efficacy when combined with other treatments. Preclinical investigations suggest that oestrogen may have proangiogenic effects by stimulating VEGF secretion, while VEGF receptor signaling may contribute to endocrine resistance (Ryden *et al*, 2005, 142). These findings would suggest that targeting both oestrogen signaling in breast cancer cells, as well as the associated tumour vasculature may provide a more effective therapeutic strategy. Preliminary results indicate that the combination of bevacizumab and letrozole is well tolerated in patients with ER+ metastatic breast cancer and resulted in stable disease in six out of 11 patients for >6months (Traina *et al*, 2005).

A number of new orally available small molecule inhibitors of the TK domain of the VEGFR are being developed. These include PTK-787 (vatalanib), a selective inhibitor of the VEGF and PDGF receptor TKs and SU11248 (sunitinib), a novel receptor TKI with potential for both direct antitumour and antiangiogenic activity by targeting kit and Fms-like TK 3(flt-3), as well as the VEGF and PDGF receptors. In a phase II trial, single-agent sunitinib showed activity in patients with advanced breast cancer (Miller *et al*, 2005). The combination of AEE788 (a dual TKI of EGFR/HER-2 and inhibitor of the VEGF receptor) and letrozole has been shown to enhance growth inhibition in ER+ breast cancer cell lines compared with either drug alone (Hauge-Evans *et al*, 2004), but this activity may be restricted to tumours that overexpress EGFR and/or HER-2. BAY43-9006 (sorafenib) is another multitargeted antiangiogenic inhibitor of both RAF kinase and the VEGFR/PDGFR-*β* signaling cascade; it is currently being investigated in a phase I/II trial in combination with anastrozole.

### Novel anti-oestrogens

As long-term treatment with an AI may cause acquired endocrine resistance and hypersensitivity to low doses of oestradiol (Martin *et al*, 2005; Santen *et al*, 2005), concomitant treatment with an additional anti-oestrogen may improve efficacy. Fulvestrant is an anti-oestrogen that leads to ER degradation and a phase II study of fulvestrant in post-menopausal women with metastatic ER+ breast cancer who had progressed on an AI demonstrated clinical benefit (PR=14%; SD=20%)(Ingle *et al*, 2006). However, preclinical data would suggest that combination rather than sequential treatment may be a superior approach. Indeed, wild-type MCF7 breast cancer cells are sensitive to fulvestrant, while LTED cells that have become hypersensitive to oestrogen require maximally suppressed oestradiol to be sensitive to fulvestrant (Dowsett *et al*, 2005a); thus, combining an AI, which decreases oestradiol to very low levels, with fulvestrant may have a synergistic effect. This would suggest that in the setting of AI resistance, there may be a rationale for adding a pure anti-oestrogen rather than switching to it and the ongoing SOFEA trial has been designed to address this question. It is a three arm randomised study of fulvestrant *vs* anastrazole+fulvestrant *vs* exemestane (a steroidal AI) in women with metastatic breast cancer who have progressed on a nonsteroidal AI.

## ONGOING AND FUTURE TRIALS

The promise of new combinations with AI has led to the development of a diverse programme of current and planned randomised, controlled trials that are summarised in Table 1. In addition to the plethora of current randomised, controlled trials, various nonrandomised clinical trials will explore the potential efficacy and safety of trastuzumab, bevacizumab, sorafenib, and erlotinib in combination with an AI as first-line or second-line therapy, and one trial will test the combination of anastrozole, fulvestrant and gefinitib in the neoadjuvant setting.

## TAILORING BREAST CANCER THERAPIES

Advanced diagnostic tools are being developed to predict sensitivity and resistance and allow for the optimisation of treatment by tailoring therapies to individual patients.

### Biomarkers

Efforts are underway to identify molecular predictors of sensitivity or resistance to particular therapies by correlating various biomarkers to treatment response. HER2 overexpression by immunohistochemistry has been well validated as a predictor of response to trastuzumab. While data suggest that HER2 upregulation may confer resistance to tamoxifen (Kurokawa *et al*, 2000), it does not appear to preclude response to AIs (Ellis *et al*, 2001). Ki67 is a marker of tumour proliferation and is becoming accepted as an early indicator of sensitivity or resistance to endocrine therapy. Indeed, changes in Ki67 as early as 2 weeks after initiation of endocrine therapy accurately predicted long-term disease-free survival in the adjuvant setting (Dowsett *et al*, 2005b). Other biomarkers such as ER and progesterone-receptor expression, pAKT and pMAPK can be correlated with changes in Ki67 to gain insight into the differential biological characteristics of tumours that are sensitive or resistant to treatment. A novel strategy is the short presurgical trial design, in which treatment-naïve patients receive a 10- to 14-day treatment with an investigative agent between the time of diagnosis and surgery, to identify *in vivo* surrogate markers of resistance or sensitivity to treatment.

### Gene profiling assays

A number of recent retrospective studies have used microarray technology to identify the gene expression profiles of tumours that are sensitive or resistant to tamoxifen (Jansen *et al*, 2005) or AIs monotherapy (MacKay *et al*, 2005). Future studies will determine whether these genomic signatures can be applied to early breast cancer patients to identify the most effective adjuvant endocrine treatment. In the future, it may be possible to predict long-term responses to treatment based on the molecular profile of a patient's pretreatment gene expression and/or changes in gene expression following a brief treatment period. In fact, preliminary data suggests that gene clusters separating pretreatment and post-treatment biopsies may be a better indicator of biological response than changes in Ki67 (MacKay *et al*, 2005).

A number of gene expression profiles have already been developed for the prognostication of women with newly diagnosed breast cancer in an effort to improve on currently available clinicopathological parameters and identify those at low-risk who may be spared adjuvant chemotherapy. Additional refinement and validation are needed before these tests can be fully integrated into patient care and large-scale trials are planned (e.g., MINDACT, TAILORx) to validate the use of these new diagnostic tools.

## CONCLUSIONS

Although tamoxifen was once the gold standard endocrine treatment for ER+ breast cancer, AIs have emerged as a viable alternative and are challenging tamoxifen as the treatment of choice for both early and advanced breast cancer in postmenopausal women with ER+disease. However a number of women invariably develop resistance to oestrogen deprivation and relapse. Several models, supported by preclinical data, have been proposed to explain the mechanisms of hormone resistance including oestrogen hypersensitivity and aberrant growth signaling pathways. This more profound understanding of the biology of resistance has led to the rational design of studies combining AIs with STIs or anti-oestrogens; these combinations offer the possibility of delaying or overcoming hormone resistance at low toxicity costs. Prior to being introduced into large clinical trials, novel combination therapies should be supported by *in vitro* models, however, healthy skepticism should be maintained since robust preclinical data may not always translate into meaningful clinical benefit, as illustrated by the recent disappointing results of combination trials with an FTI or an mTOR inhibitor. These observations further underscore the crucial need for early phase I/II trials to include parallel biological studies examining the association of protein or gene expression profiles with treatment response as enrolling an unselected population into large phase III trials may dilute the potential beneficial effects of a specific combination for a subset of patients. In the future, breast cancer therapy may be tailored to each patient to maximise benefits. This individualised treatment will only be possible by identifying and validating biomarkers that accurately predict which patients are most likely to benefit from various combined endocrine and STI therapies and the results of the ongoing combination trials will define whether regimens combining AIs with novel targeted agents should become an integral part of the next generation of breast cancer treatment.

## Figures and Tables

**Table 1:** Current, recently closed and planned randomized, controlled trials of signal transduction inhibitors and antiestrogens in combination with AI in breast cancer

**Trial name/sponsor**	**Treatments**	**Study phase**	**Patients (*N*)**	**Population**
*Monoclonal antibodies*				
B016216/Roche	ANA±trastuzumab	II/III	202	First/second line; ER+/HER-2+
CFEM 345C2403/Novartis	LET±trastuzumab	IV	NR	First/second line; ER+/HER-2+
EU-20527	LET±trastuzumab	II	30–40	Second line; ER+/HER-2+
Pharmacia-NU-01B4	EXE±trastuzumab	II	18–60	First/second line; ER+/HER-2+
CC#037518	LET+bevacizumab	II	NR	First line
UAB 0467	LET+bevacizumab	II	25	Neoadjuvant
				
*Tyrosine kinase inhibitors*				
Trial 223 (Europe)	ANA±gefitinib	II	185	Neoadjuvant
CTRC, San Antonio	ANA±gefitinib	II	78	Second line
AstraZeneca 0713	ANA±gefitinib	II	174	First/second line
EORTC 10021	ANA±gefitinib	II	108	First line
ECOG 4101	ANA+gefitinib+*vs* FUL+gefitinib	II	106	First/second line
GSK EGFR30008	LET±lapatinib	III	760	First/second line
VICC BRE 0303	LET±erlotinib	II	150	Second line
				
*Farnesyl transferase inhibitors*				
JJPRD R115777-INT-22	LET±tipifarnib	II	108	Second line
CWRU-JJPR-1102 (Ireland Cancer Center)	LET±tipifarnib	II	120	Second line
SCH 66336	ANA±lonafarnib	II	110	First line
UCLA-0403073-01 (JCCC/NCI)	ANA±lonafarnib	II	110	First line
				
*mTOR inhibitors*				
Wyeth Ayerst	LET±temsirolimus	II	90	First/second line
Ireland Cancer Center	LET±temsirolimus	II	108	Second line
Wyeth Ayerst 3066A1-303	LET±temsirolimus	III	1236	First line
Novartis	LET±everolimus	II/III	600	First line
Novartis CRAD001C223	LET±everolimus	II	212	Neoadjuvant
				
*Antiestrogens*				
SOFEA	ANA+FUL *vs* FUL *vs* EXE	III	750	Postnonsteroidal AI
FACT	ANA±FUL	III	558	First line
SWOG-S0226	ANA±FUL	III	690	First line
FIRST	ANA±FUL	II	200	First line
D6997C00057	ANA±FUL	II	120	Neoadjuvant
CAT Study	LET *vs* atamestane+toremifene	III	842	First line
OSU-0494	EXE±FUL	II	40	First/Second line
EFECT	EXE *vs* FUL	III	660	Postnonsteroidal AI

*Note*: TAM=tamoxifen; ANA=anastrozole; LET=letrozole; FUL=fulvestrant; EXE=exemestane; NR=not reported; ER+=oestrogen receptor-positive; EGFR=epidermal growth-factor receptor; HER-2+=human epidermal growth-factor receptor 2; AI=aromatase inhibitor.

CALGB=Cancer and Leukemia Group B.

CFEM=Clinical (Research with) Femara.

CTRC=Cancer Therapy & Research Center.

ECOG=Eastern Cooperative Oncology Group.

EFECT=The Evaluation of Faslodex and Exemstane Clinical Trial.

EORTC=European Organization for Research and Treatment of Cancer.

FACT=The Faslodex and Arimidex in Combination Trial.

JCCC/NCI=Jonsson Comprehensive Cancer Center/National Cancer Institute.

J&J PRD=Johnson & Johnson Pharmaceutical Research & Development.

SCH=Schering Plough.

SOFEA=Study of Faslodex *vs* Exemestane with or without Arimidex.

SWOG=Southwest Oncology Group.

UAB=University of Alabama.

UCLA=University of California at Los Angeles.

VICC=Vanderbilt-Ingram Cancer Center.
